# Virgin Olive oil Authenticity Assays in a Single Run Using Two-Dimensional Liquid Chromatography-High Resolution Mass Spectrometry

**DOI:** 10.1021/acs.analchem.4c03678

**Published:** 2024-10-15

**Authors:** Irene Caño-Carrillo, Bienvenida Gilbert-López, Cristina Ruiz-Samblás, Antonio Molina-Díaz, Juan F. García-Reyes

**Affiliations:** † Analytical Chemistry Research Group, Department of Physical and Analytical Chemistry, University of Jaén, Campus Las Lagunillas, 23071 Jaén, Spain; ‡ University Research Institute for Olives Grove and Olive Oil, University of Jaén, Campus Las Lagunillas, 23071 Jaén, Spain

## Abstract

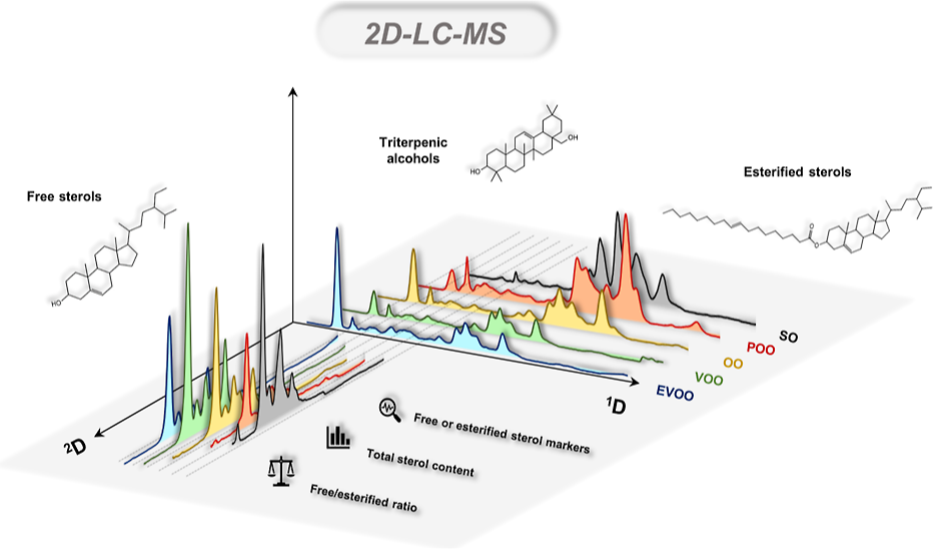

Sterols and triterpenic alcohol analyses are one of the officially established parameters for assessing the authenticity of virgin olive oil (VOO). Most of the applications described for sterol analysis, including the official method, only allow the determination of the total sterol content but not its distribution in free or esterified form. This work proposes a two-dimensional liquid chromatography/high-resolution mass spectrometry (2D-LC-HRMS) method for the simultaneous analysis of triterpenic alcohols, free sterols and steryl esters. A reversed phase liquid chromatography (RPLC)–RPLC coupling was performed through a multiple heart-cutting interface equipped with an active solvent modulation (ASM) valve. Additionally, a selection valve was coupled to the 2D-LC system, allowing the simultaneous data acquisition from both dimensions in a single analysis. A simplified sample treatment based on solid phase extraction was also proposed to avoid tedious steps such as saponification or derivatization before gas chromatography analysis. To evaluate the content of these compounds in different olive oil categories, the proposed 2D-LC-HRMS system was applied to a set of samples from different commercial olive oil categories (extra virgin olive oil, virgin olive oil, olive oil, pomace olive oil) and sunflower oil. The results revealed significant differences in the distribution of free and esterified sterols of the analyzed samples, highlighting the free/esterified sterol ratio as a powerful tool to unveil olive oil fraud practices and fat manipulation.

Virgin olive oil (VOO) is one of the most valuable vegetable oils due to its distinctive organoleptic properties and nutritional value.^1^ According to data from the International Olive Council (IOC), olive oil prices at mills have reached maximum levels in the last two seasons, rising by nearly 200% compared to the previous season.^2^ As a result of this price increase, olive oil has become one of the main targets of fraudulent activities in the agricultural sector.^3^ To ensure the quality and authenticity of olive oil, several analytical methods are established in European Union regulations and IOC standards,^1^ including the analysis of sterols and triterpenic alcohols.^4^ These compounds are part of the unsaponifiable fraction, which represents 2% of the total weight of the oil.^5^ This minor fraction content and composition is of great importance not only to verify the authenticity of olive oil, but also to evaluate other aspects, such as their biological activity and positive impact on human health,^6^ as well as their potential for discrimination of geographical oil origin^7^ or varietal differentiation.^8^

Most methods described in the literature for the determination of sterols and triterpenic alcohols are based on gas chromatography (GC).^9−12^ The official EU method also uses GC after a previous process of saponification, separation, and derivatization.^13^ However, the lack of selectivity of this detection system and the complexity of the matrix analyzed make GC-flame ionization detection (GC-FID) methods not always reliable and robust enough to analyze sterols in oil samples. Therefore, the use of GC coupled to mass spectrometry (GC–MS) has also been applied for this type of analysis.^14−16^ Liquid chromatography (LC) has also been used as an alternative to GC, particularly for the analysis of thermally unstable compounds such as sterols.^17^ LC was coupled to different detection methods such as diode array detection|diode array detector (DAD),^18^ evaporative light scattering^19^ or MS^7,20,21^ typically using atmospheric pressure chemical ionization (APCI), which offers better results than electrospray ionization (ESI) due to the nonpolar nature of these compounds.^22^

An important consideration is that, in vegetable oils, sterols can be found in free form or esterified with fatty acids. The distribution of fatty acids available for esterification varies greatly between oils.^12^ However, the official method and most of the applications mentioned above are based on the saponification process. Therefore, only total sterol content can be determined, regardless of whether the sterols were originally in free or esterified form. This represents a significant loss of information that could be particularly useful for distinguishing oils with similar sterol profiles, characterization purposes or identifying adulterations.^10^ Only a few studies have attempted to cover the simultaneous analysis of free and esterified sterols (2-step indirect determination), using solid phase extraction (SPE) to achieve the separation of both fractions and employing GC-FID^23^ or GC–MS.^24^

More complex systems have also been used to analyze sterols in both free and esterified forms. The first attempt was proposed by Grob,^25^ who developed an LC–GC-FID method to analyze free and esterified sterols. Other authors later followed a similar strategy.^26,27^ Multidimensional strategies based on comprehensive GC (GC×GC) have also been proposed for the simultaneous determination of minor compounds in edible oils.^28^ Using two different separation mechanisms increases the range of compounds that can be separated in a single analysis, making two-dimensional (2D) chromatography a powerful tool for fingerprinting and characterizing the unsaponifiable oil fraction.^29,30^ However, these 2D methods do not avoid the tedious process of derivatization as a preliminary step to GC analysis and the possibility of subtle chemical changes during the reaction.

The present study aimed to develop a derivatization-free method for the simultaneous analysis of triterpenic alcohols and sterols, both in their free and esterified form, trying to overcome some of the drawbacks found in the published methods for oil analysis. To this end, the use of two-dimensional LC (2D-LC) is proposed as an alternative to the GCxGC separations described in the literature, but avoiding the derivatization process. Moreover, to simplify the sample treatment and to circumvent saponification, SPE was used to extract directly the sterols in their original form from the oil samples. The proposed strategy was to analyze the steryl esters in the ^1^D and transfer the void volume, containing the free sterols and triterpenic alcohols, to the ^2^D in a single cut. In addition, the developed approach allowed the acquisition of both MS dimensions in a single data file by coupling a selection valve to the 2D-LC setup. The system was evaluated with a mixture of 21 compounds, including triterpenic alcohols, free sterols and steryl esters. The determination of the compounds established in the official IOC method was studied on 25 oil samples, including olive oil of different commercial categories and sunflower oil. In summary, this paper presents for the first time the development of a 2D-LC-HRMS method for the simultaneous analysis of triterpenic alcohols, free and esterified sterols in oil samples. The main features sought with this approach are to obtain thorough information from the oil sample by determining the distribution of free or esterified sterols, as well as simplifying the procedure by skipping time-consuming steps such as saponification and derivatization.

## Experimental Section

### Chemicals and Reagents

Sterols and cholesteryl esters standards were acquired from Sigma-Aldrich (Steinheim, Germany). Three-noncommercially available-steryl esters (β-sitosteryl oleate, stigmasteryl oleate and sitostanyl oleate) were prepared according to Barnsteiner^31^ with modifications in the initial reagent amount. Details of the synthesis are provided in the Supporting Information. HPLC-grade isopropanol, acetonitrile, water, methanol, ethanol, chloroform, *n*-hexane, ethyl acetate, diethyl ether, methyl *tert*-butyl ether, and potassium hydroxide were purchased from Merck (Darmstadt, Germany). Formic acid was acquired from Scharlab (Barcelona, Spain). Silica SPE cartridges (1000 mg, 6 mL) were obtained from Macherey-Nagel (Düren, Germany). Individual stock solutions (Table 2) (ca. 500 mg L^–1^ each) were prepared in ethanol for sterols or chloroform/methanol (1:1, v/v) for cholesteryl esters. They were stored at −20 °C until use.

### Samples

25 oil samples purchased in local markets in Spain were selected for this study: 5 samples from four different olive oil categories [extra virgin olive oil (EVOO), virgin olive oil (VOO), olive oil (OO), pomace olive oil (POO)] and 5 samples of sunflower oil (SO). The purpose of analyzing this type of sample was to determine the distribution of free and esterified sterols in the different olive oil categories. In addition, SO was selected for analysis since it is one of the most common vegetable oils used for adulteration due to its lower cost.

### Sample treatment

Sterols and steryl esters were extracted from oil samples using a solid-phase extraction protocol with silica SPE cartridges adapted from Cunha.^24^ Briefly, 0.25 g of oil sample was weighed in a test tube and diluted with 1 mL of *n*-hexane/ethyl acetate (90:10, v/v). The oil sample solution was loaded into the SPE cartridge previously conditioned with 2 × 5 mL of *n*-hexane. Then, an aliquot of 2.5 mL of *n*-hexane/ethyl acetate (90:10, v/v) was passed through the cartridge. This first eluate containing the esterified fraction was evaporated by a gentle nitrogen stream and redissolved with 1 mL of ACN/IPA (55:45, v/v). After eluting the steryl ester fraction, 5 mL of *n*-hexane/ethyl acetate (90:10, v/v) and 3 × 5 mL ethanol/diethyl ether/*n*-hexane (50:25:25, v/v) were employed to elute the free sterols. This second eluate was evaporated using a rotary evaporator and redissolved with 1 mL of ACN/IPA (55:45, v/v). Finally, both extracts were combined in a single solution with different dilution factors (1:10 for steryl esters and 1:100 for free sterols).

### 2D-LC Setup

Heart-cutting 2D-LC analyses were carried out on an Agilent 1290 Infinity ultra high-performance liquid chromatography system (Agilent Technologies, Santa Clara, CA) equipped with an autosampler and a DAD, acquiring at 200 nm with a sampling rate of 20 Hz. An additional Agilent 1290 Infinity II LC pump was coupled to perform the 2D separation. The first and second dimensions were interfaced by a 5-position/10-port active solvent modulation (ASM) valve connected via two 1.9 μL stainless steel [stainless steel transfer (SST)] transfer capillaries (170 × 0.12 mm) to two 6-position/14-port multiple heart-cutting (MHC) valves, each containing six 180 μL SST sampling loops (1870 × 0.35 mm). In addition, the 2D-LC system (Figure S1) was coupled to a time-of-flight (TOF) mass spectrometer (Agilent 6220 accurate mass TOF, Agilent) equipped with an APCI source operated in the positive ion mode, using the following conditions: capillary voltage, 3000 V; nebulizer pressure, 40 psi; drying gas, 5 L min^–1^; gas temperature, 250 °C; vaporizer temperature, 350 °C; corona current, 4 μA; skimmer voltage, 65 V; octopole rf, 250 V; fragmentor voltage, 120 V; mass range, *m*/*z* 100–1000 Da. Data acquisition of both dimensions in MS was achieved by coupling a 2-position/6-port column selection valve before the mass spectrometer, allowing to control the ^1^D or ^2^D effluent inlet depending on the time of analysis. Agilent OpenLab CDS Chemstation software (version A.01.04) and Agilent Mass Hunter Data Acquisition software (version B.04.00) were used to control the 2D-LC system and acquire the MS data, respectively. The LC–LC data were visualized and processed with Agilent Mass Hunter Qualitative Analysis software (version 10.0). The Statistical Package for the Social Science software was used for the statistical analysis.

### Chromatographic Conditions

Nine different columns were evaluated for the analysis of triterpenic alcohols, free sterols and steryl esters: (1) Zorbax Eclipse Plus C18 (150 mm × 4.6 mm, 1.8 μm), (2) Zorbax Eclipse XDB-C18 (100 mm × 4.6 mm, 1.8 μm), (3) Kinetex C18 (150 mm × 4.6 mm, 2.6 μm), (4) Zorbax Bonus RP (50 mm × 2.1 mm, 1.8 μm), (5) InfinityLab Poroshell 120 EC-C18 (50 mm × 2.1 mm, 1.9 μm), (6) InfinityLab Poroshell 120 PFP (100 mm × 2.1 mm, 1.9 μm), (7) SB-Phenyl (100 mm × 2.1 mm, 1.8 μm), (8) Zorbax Eclipse Plus C18 (100 mm × 3.0 mm, 1.8 μm), (9) Zorbax Eclipse Plus C18 (50 mm × 2.1 mm, 1.8 μm). Stepwise optimization of each dimension was initially accomplished, evaluating the mobile phase composition, flow rate, and elution gradient as main parameters for the different columns. Once the preliminary conditions were obtained, both dimensions were coupled. The final chromatographic conditions of the 2D-LC approach are summarized in Table 1.

**Table 1:** Optimized Chromatographic Conditions for 2D-LC Analysis

	^1^D			^2^D		
**column**	Zorbax Eclipse Plus C_18_ (50 mm × 2.1 mm, 1.8 μm)			InfinityLab Poroshell 120 EC-C_18_ (50 mm × 2.1 mm, 1.9 μm)		
						
**mobile phase**	A: acetonitrile			A: water (0.1% formic acid)		
	B: isopropanol			B: methanol (0.1% formic acid)		
						
**flow**	0.5 mL/min			0.5 mL/min		
						
**gradient program**	time (min)	% B		time (min)	% B	
	0	45		0	2	
	9	48		3.32	2	
	9.1	45		4	90	
	12	45		5	93	
				9	94	
	post-time: 3 min			10	100	
				11	100	
	injection volume: 20 μL					
	DAD detector 200 nm (20 Hz)			post-time: 4 min		
				loops: 180 μL		
				ASM phase: 3.32 min		
						
**fraction sampling and 2D-LC analysis**	cut	start (min)	sampling time (min)	selection valve		MS detection
				time (min)	pos	
	#1	0.60	0.36	0–8.5	1	^1^D
				8.5–15	2	^2^D

## Results and Discussion

### Preliminary Studies and Stepwise Optimization of the ^1^D and ^2^D Separations

The separation of triterpenic alcohols and free/esterified sterols by LC has not been extensively studied. First, a preliminary evaluation of the combined analysis of triterpenic alcohols, free sterols and steryl esters by one-dimensional LC was attempted. Up to seven columns using different mobile phase compositions and gradients were tested (see Supporting Information for details). All these preliminary experiments revealed the need to use different separation conditions for free and esterified sterols. Therefore, since this study aimed to analyze both fractions simultaneously, the use of 2D-LC was proposed due to its ability to implement complementary selectivities in a single analysis. The preliminary tests showed that under the unique conditions that allowed the separation of the steryl esters, free sterols and triterpenic alcohols eluted together close to the void volume. Therefore, the proposed strategy was to analyze the esters in the ^1^D and transfer in a single cut the free fraction to the ^2^D for their analysis under optimal conditions.

The optimization of the ^1^D was performed using all commercially available (8) cholesteryl esters standards. Different tests were performed with column 1 (Zorbax Eclipse Plus C18) using the two-dimensional configuration (autosampler—^1^D column—DAD—ASM valve—MS) with 40 μL loops installed in the MHC valves. Different ACN/IPA gradients with flow rates from 0.2 to 0.5 mL/min were evaluated, yielding the best separation with a linear gradient from 45% B to 48% B in 9 min (0.5 mL min^–1^ flow rate). Cholesteryl esters were eluted according to fatty acid chain length and the number of unsaturations: C10:0, C18:3, C18:2, C14:0, C18:1, C16:0, C18:0, C22:1. This elution order for cholesteryl esters can be extrapolated to the rest of the steryl ester series.^32^

**Figure 1:**
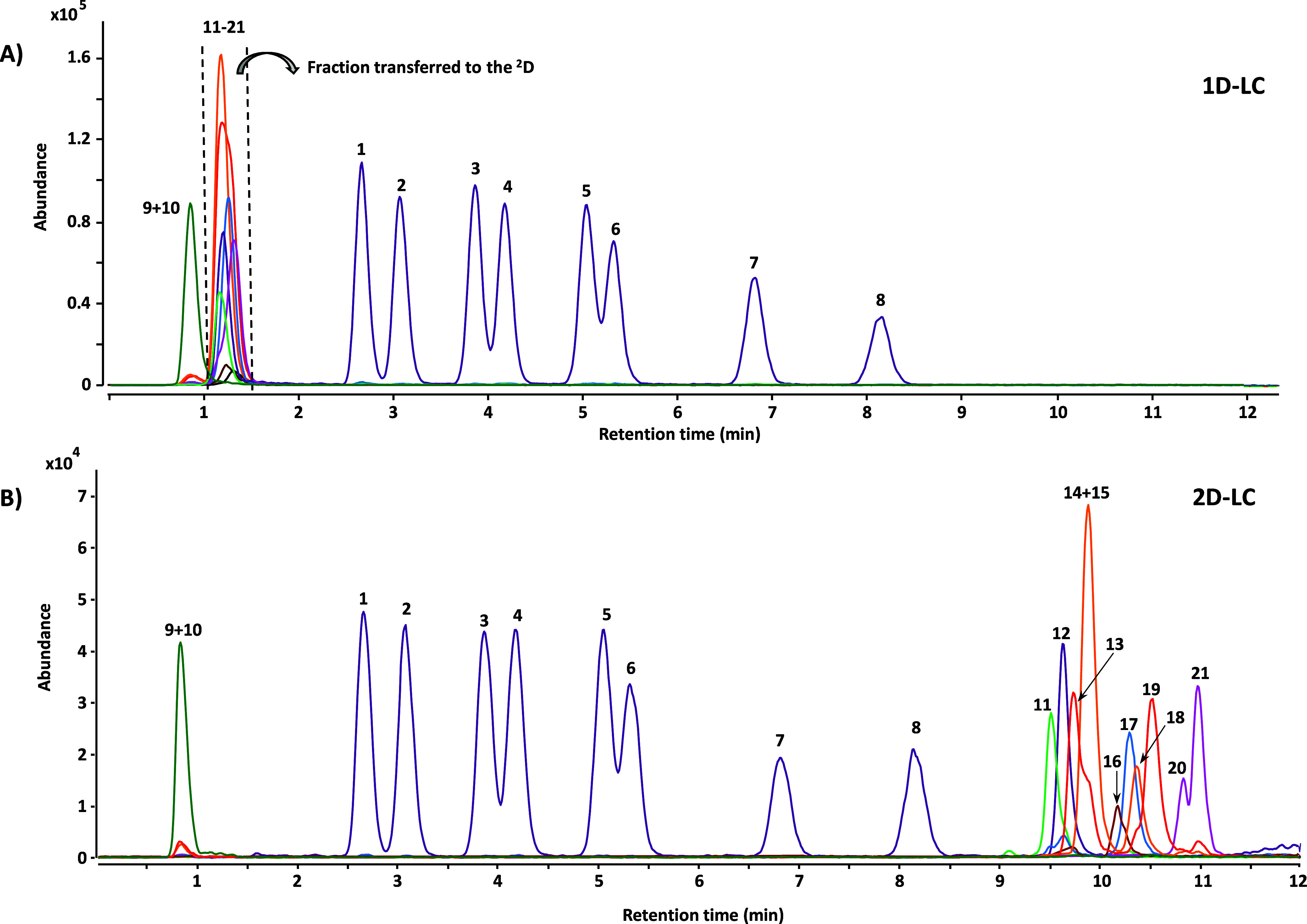
(A) ^1^D-LC chromatogram obtained with optimal chromatographic conditions and all standards mixture. (B) 2D-LC chromatogram with simultaneous visualization of the ^1^D and ^2^D using the selection valve approach. A standard mixture containing 1 mg L^–1^ for the ^1^D analytes and 100 μg L^–1^ for the ^2^D analytes was used. The peaks have been numbered as follows: 1. cholesteryl decylate; 2. cholesteryl linolenate; 3. cholesteryl linoleate; 4. cholesteryl myristate; 5. cholesteryl oleate; 6. cholesteryl palmitate; 7. cholesteryl stearate; 8. cholesteryl erucate; 9. erythrodiol; 10. uvaol; 11. brassicasterol; 12. cholesterol; 13. lupeol; 14. fucosterol; 15. Δ^5^-avenasterol; 16. cholestanol; 17. campesterol; 18. stigmasterol; 19. β-amyrin; 20. Δ^7^-stigmastenol; 21. β-sitosterol.

Once the separation of the cholesteryl esters was optimized, an assay including the free sterol and triterpenic alcohols was carried out to evaluate their behavior in the ^1^D (Table 2). As expected, all free analytes eluted together in the void volume. However, under these conditions, the void volume was too broad (≈1 min) to transfer the whole free fraction to the ^2^D in a single cut, as the maximum sampling time was 0.08 min. To reduce the void volume, some experiments were performed with the same stationary phase (Zorbax Eclipse Plus C18), but decreased the length and inner diameter of the column. For this purpose, column 8 (100 mm × 3.0 mm, 1.8 μm) and column 9 (50 mm × 2.1 mm, 1.8 μm) were evaluated. A narrower void volume was obtained with the shortest column, so finally, column 9 and 180 μL sampling loops were selected to perform the 2D-LC analysis. The initial strategy was to transfer to the ^2^D the erythrodiol/uvaol peak (Figure 1A) together with the rest of the free sterols. However, these triterpenic alcohols eluted at a different retention time than the rest of the free sterols/triterpenic alcohols. This could be attributed to the presence of two alcohol groups unlike all other free sterols and triterpenic alcohols. Therefore, it was decided to keep these two compounds in the ^1^D.

**Table 2:** Overview of the 2D-LC HRMS Identification Features of the Selected Chemicals

#	compound	separation	elemental composition	selected ion	*m*/*z* theoretical	retention time (min)
1	cholesteryl decylate	^1^D	C_37_H_64_O_2_	[M-decanoid acid]^+^	369.3516	2.65
2	cholesteryl linolenate	^1^D	C_45_H_74_O_2_	[M-linolenic acid]^+^	369.3516	3.07
3	cholesteryl linoleate	^1^D	C_45_H_76_O_2_	[M-linoleic acid]^+^	369.3516	3.86
4	cholesteryl myristate	^1^D	C_41_H_72_O_2_	[M-myristic acid]^+^	369.3516	4.17
5	cholesteryl oleate	^1^D	C_45_H_78_O_2_	[M-oleic acid]^+^	369.3516	5.05
6	cholesteryl palmitate	^1^D	C_43_H_76_O_2_	[M-palmitic acid]^+^	369.3516	5.31
7	cholesteryl stearate	^1^D	C_45_H_80_O_2_	[M-stearic acid]^+^	369.3516	6.80
8	cholesteryl erucate	^1^D	C_49_H_86_O_2_	[M-erucicacid]^+^	369.3516	8.14
9	erythrodiol	^1^D	C_30_H_50_O_2_	[M + H–H_2_O]^+^	425.3778	0.83
10	uvaol	^1^D	C_30_H_50_O_2_	[M + H–H_2_O]^+^	425.3778	0.83
11	brassicasterol	^2^D	C_28_H_46_O	[M + H–H_2_O]^+^	381.3516	9.50
12	cholesterol	^2^D	C_27_H_46_O	[M + H–H_2_O]^+^	369.3516	9.62
13	lupeol	^2^D	C_30_H_50_O	[M + H–H_2_O]^+^	409.3829	9.73
14	fucosterol	^2^D	C_29_H_48_O	[M + H–H_2_O]^+^	395.3672	9.88
15	Δ^5^-avenasterol	^2^D	C_29_H_48_O	[M + H–H_2_O]^+^	395.3672	9.88
16	cholestanol	^2^D	C_27_H_48_O	[M + H–H_2_O]^+^	371.3672	10.17
17	campesterol	^2^D	C_28_H_48_O	[M + H–H_2_O]^+^	383.3672	10.29
18	stigmasterol	^2^D	C_29_H_48_O	[M + H–H_2_O]^+^	395.3672	10.36
19	β-amyrin	^2^D	C_30_H_50_O	[M + H–H_2_O]^+^	409.3829	10.52
20	Δ^7^-stigmastenol	^2^D	C_29_H_50_O	[M + H–H_2_O]^+^	397.3829	10.83
21	β-sitosterol	^2^D	C_29_H_50_O	[M + H–H_2_O]^+^	397.3829	10.97

A standard solution of 9 free sterols and 4 triterpenic alcohols was used to optimize the ^2^D separation. The first step was to analyze the complete mixture of compounds with the conditions that provided the best results in preliminary tests: (5) InfinityLab Poroshell 120 EC-C18 column (H_2_O + 0.1% FA/MeOH + 0.1% FA), (6) InfinityLab Poroshell 120 PFP column (H_2_O + 0.01% AA/ACN) and (7) SB-Phenyl column (H_2_O + 0.1% FA/MeOH + 0.1% FA). The main difficulty in the separation was the coelution of several isobaric species, erythrodiol/uvaol, fucosterol/Δ^5^-avenasterol and β-sitosterol/Δ^7^-stigmastenol. Additional MS/MS experiments can differentiate these pairs based on characteristic fragmentation.^33,34^ In addition, the criteria established by the European Regulation for olive oil and its sterol content in the different categories do not require the separation of all the species.^35^ This regulation includes fucosterol and Δ^5^-avenasterol as part of the so-called apparent β-sitosterol. Moreover, it establishes the erythrodiol and uvaol content as the sum of both triterpenic alcohols. Taking all of these considerations in mind, the InfinityLab Poroshell 120 EC-C18 column was selected for the ^2^D analysis as it was able to separate Δ^7^-stigmastenol and β-sitosterol, the latter being the most abundant sterol in olive oil. Although the PFP column also resolved Δ^7^-stigmastenol and β-sitosterol, it was discarded as it yielded coelution of Δ^7^-stigmastenol, campesterol, and stigmasterol, used as markers of the adulteration of olive oils with vegetable oils.^23^ The final optimal separation conditions with the InfinityLab Poroshell 120 EC-C18 selected are shown in Table 1.

### Heart-Cutting (RPLC–RPLC) 2D-LC-HRMS Method Performance

Once the separations were individually optimized, both dimensions were coupled through an MHC interface equipped with an ASM valve (Figure S1), the latter being responsible for eliminating solvent incompatibility. Despite working with a reversed phase liquid chromatography (RPLC)–RPLC system, it was necessary to use the ASM valve because the free sterol/triterpenic alcohol fraction solvent composition (ACN/IPA) taken from the ^1^D had a higher eluent strength than the ^2^D mobile phase (H_2_O/MeOH). For this purpose, several ASM dilution times were tested, achieving the best refocusing of the analytes in the ^2^D column head and, thus, the best peak shape and sensitivity, with an ASM time of 3.32 min (equivalent to 6 ASM cycles).

One of the objectives of this study was to acquire the MS data from both dimensions in a single file. A selection valve before the MS was included in the 2D-LC assembly to control the inlet of the ^1^D or ^2^D flow to the MS, depending on the analysis time. The sequence of steps is detailed in Table 1. At 8.5 min, just after the ^1^D analysis was finished and the MS data acquired, the selection valve was switched to position 2, so that the ^2^D effluent was then diverted to the MS. The selection valve was already kept in position 2 until the end of the analysis. Hence, it was possible to acquire both dimensions in a single run by MS without losing any information. The proposed strategy is streamlined since it is possible to analyze both dimensions in only 15 min. A typical chromatogram resulting from the two-dimensional separation is shown in Figure 1B.

Compound annotation was performed by comparison of retention time and accurate mass measurements for those analytes for which standards were available (Table 2). The [M + H–H_2_O]^+^ fragment from the neutral loss of water was obtained as a base peak for sterols and triterpenic alcohols via APCI-MS analysis. For the dialcohols erythrodiol and uvaol, the [M + H–2H_2_O]^+^ ion was also detected, although with lower intensity than the monodehydrated ion. Steryl esters identification was made using the [M – fatty acid + H]^+^ fragment, allowing discrimination between those esters with different sterol nuclei. Although the development of the proposed method was carried out with a limited number of compounds (Table 2), compound annotation of the sterols included in the official IOC method,^13^ either in their free or esterified form were performed using edible oil samples with a characteristic sterol composition and additional analyses of the saponified samples using the reference GC method. We made use of two different oil samples, expected to have different sterol and triterpenic alcohol profiles, so that they could be identified by comparing the differences between both oils. For this purpose, a VOO and a SO sample were selected and analyzed using the 2D-LC-MS method. In addition, both samples were subjected to the procedure described in the official method for the determination of total sterols and triterpenic alcohols.^13^

Calibration curves of the 21 available standards were prepared (Supporting Information). Different concentration ranges were used for each dimension: 10–500 μg L^–1^ for cholesteryl esters, erythrodiol and uvaol (first dimension), and 100–5000 μg L^–1^ for free sterols, lupeol and β-amyrin (second dimension). Good linearity was obtained for all compounds, with regression coefficients above 0.99. To evaluate the interday precision, a mixture of standards at an intermediate concentration of 1000 μg L^–1^ for the ^1^D compounds and 100 μg L^–1^ for the ^2^D compounds was analyzed over 3 weeks (*n* = 6). These results were in the range of 13–19%. Relative standard deviation (RSD) (*n* = 3) values to assess intraday reproducibility ranged from 0.4 to 8.2%. Limits of quantitation (LOQs) were established as the minimum quantifiable analyte concentration for a signal-to-noise ratio (S/N) = 10. The LOQs for the ^2^D analytes were an order of magnitude lower than those for the ^1^D analytes. This can be attributed to the fact that, in the second dimension, only a specific portion of the matrix containing the analytes of interest is transferred to the MS, while the rest of the ^1^D effluent is fully analyzed. Consequently, most of the matrix interferents reach the MS during the ^1^D analysis, thereby reducing the matrix effect in the ^2^D and enhancing the sensitivity.^36^ Anyhow, the compounds targeted in this application are not found at low concentration levels in oil samples, so very stringent LOQs would not be necessary whatsoever. Further sensitivity enhancement could be achieved using MS/MS (MRM) detection.

After the annotation of the main species, a semiquantification of free and esterified sterols was carried out. For this purpose, a total of 25 samples were selected: 5 samples from four different olive oil categories (EVOO, VOO, OO, POO) and 5 samples of SO. The results obtained by the 2D-LC–MS method are shown in Table 3. Semiquantification of esters was performed with the calibration curve of cholesteryl oleate. For free sterols for which no standard was available, the semiquantification was carried out using the calibration curve of the most similar sterol (same *m*/*z* value).

**Table 3:** Determination of Free and Esterified Sterols in EVOO, VOO, OO, POO, and SO Samples by 2D-LC-MS. RSD Values are Expressed as the Variation of Sterols in the Different Samples Analyzed for Each Set of Oil

					RSD (%) (*n* = 5)		
oil sample		free (mg/kg)	esterified (mg/kg)	free/esterified ratio	free	esterified	total sterols (mg/100 g)
EVOO	M1	1423	282	5.0	8.2	28.6	170.5
	M2	1299	421	3.1			172.0
	M3	1614	219	7.4			183.3
	M4	1381	229	6.0			161.0
	M5	1395	267	5.2			166.2
VOO	M6	2436	360	6.8	29.9	49.6	279.6
	M7	1832	215	8.5			204.7
	M8	2593	299	8.7			289.2
	M9	1955	610	3.2			256.5
	M10	1088	196	5.5			128.4
OO	M11	897	542	1.6	20.3	20.6	143.9
	M12	1393	719	1.9			211.2
	M13	1579	734	2.1			231.3
	M14	1559	963	1.6			252.2
	M15	1530	809	1.9			233.9
POO	M16	954	1298	0.7	21.9	35.1	225.2
	M17	992	1466	0.7			245.8
	M18	1512	1475	1.0			298.7
	M19	1357	2821	0.5			417.8
	M20	996	2282	0.4			327.8
SO	M21	1664	1575	1.1	20.0	7.3	323.9
	M22	2200	1344	1.6			354.4
	M23	1605	1546	1.0			315.1
	M24	2511	1642	1.5			415.3
	M25	2375	1561	1.5			393.6

**Figure 2:**
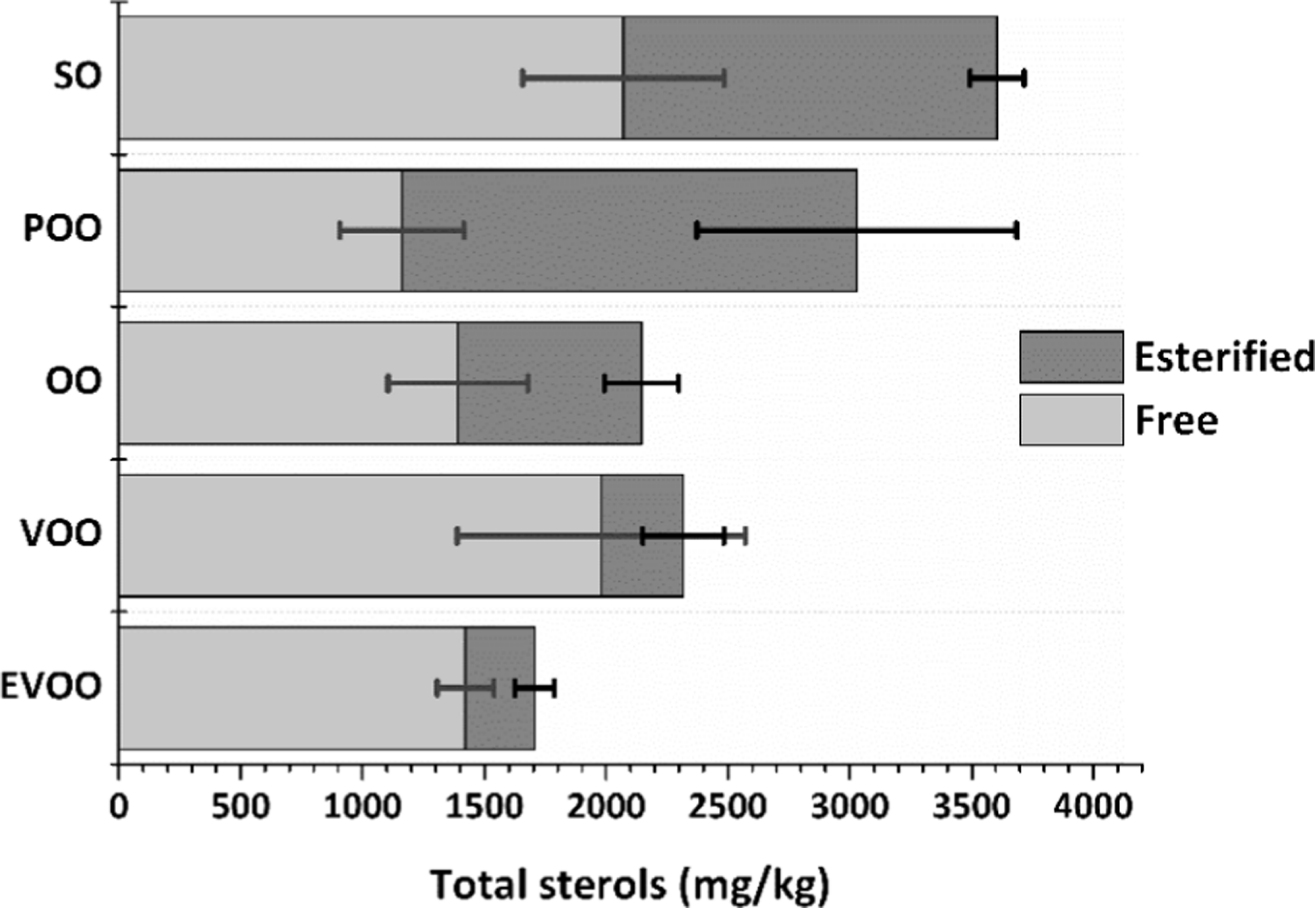
Comparison of the mean value (*n* = 5) for the total content of free and esterified sterols in the different oil samples studied.

Total sterols were obtained as the sum of free sterols and esterified sterols. Different content of total sterols was obtained for the four categories of olive oil, ranging from 1610 to 1833 mg/kg for EVOO, between 1284 and 2892 mg/kg for VOO, from 1439 to 2522 mg/kg for OO, and the highest content for POO with values between 2252 and 4178 mg/kg. These values are within the current regulation for total sterol content in OO and POO oils.^4^ Additionally, the lower total sterol content obtained from olive oils compared to SO is consistent with the literature.^16^ Significant differences were also noticed in olive oils when focusing on the free and esterified sterol fractions (Figure 2). A higher content of esterified sterols was obtained for OO and POO (lower quality oils) compared to the higher quality categories, such as EVOO and VOO, for which the esterified levels were below 1000 mg/kg in all samples analyzed. These variations are also evidenced when comparing the free/esterified ratio of each category, with a range between 1.6 and 2.1 for OO and 0.4–1.0 for POO, while for EVOO and VOO, values between 3.1 and 7.4 and 3.2–8.7 were obtained, respectively. The higher esterified content in OO and POO could be justified by the effect of temperature on the esterification process of sterols during the refining process.^12^ Regarding SO, a higher content of total sterols was quantified compared to olive oils, with a greater proportion of the esterified fraction in contrast to the higher quality olive oil categories (EVOO and VOO). These results are consistent with those described in the literature for this oil type.^10^

It is worth noting that a similar esters range was obtained for all the SO samples studied (RSD % = 7.3%), while the results were more scattered for the free fraction (RSD % = 20.0%). However, the opposite scenario was found for EVOOs. As stated above, the main fraction was free sterols, which showed less variation in the analyzed samples (RSD % = 8.2%) than the esterified fraction (RSD % = 28.6%). Therefore, it highlighted the importance of analyzing free and esterified fractions since each oil has its characteristic pattern. The information provided by each of them could be used as a marker for EVOO adulteration by sunflower oils or for characterization purposes.

Furthermore, a statistical analysis of the results presented in Table 3 was performed. To this end, one-way analysis of variance and a post hoc Tukey’s test (*p* < 0.05) were used to determine the significant differences according to the free/esterified sterol ratio and the total sterol content. As shown in Table 4, the free/esterified sterol ratio revealed significant differences between virgin olive oils (EVOO and VOO) and refined oils (OO, POO, SO). This emphasizes the importance of considering this free/esterified ratio parameter, given that these mixtures are typically reported in olive oil adulteration. Additionally, the total sterol content also showed significant differences between olive oils and other vegetable oils, with SO being considered in this study. These results would allow to confirm the discriminatory potential of the developed approach.

**Table 4:** Mean Values of Total Sterol Content and Free/Esterified Sterol Ratio for the Different Types of Oils Analyzed^a^

	EVOO	VOO	OO	POO	SO
free/esterified sterol ratio	5.3 ± 1.6^a^	6.5 ± 2.3^a^	1.8 ± 0.2^b^	0.7 ± 0.2^b^	1.3 ± 0.3^b^
total sterols (mg/100 g)	170.6 ± 8.3^α^	231.7 ± 66.4^α,β^	214.5 ± 42.1^α,β^	303.1 ± 76.1^β,γ^	360.5 ± 3.4^γ^

a Values are expressed as mean ± standard deviation. Different superscript letters within a row indicate significantly different values (*p* < 0.05) by Tukey’s test.

**Figure 3:**
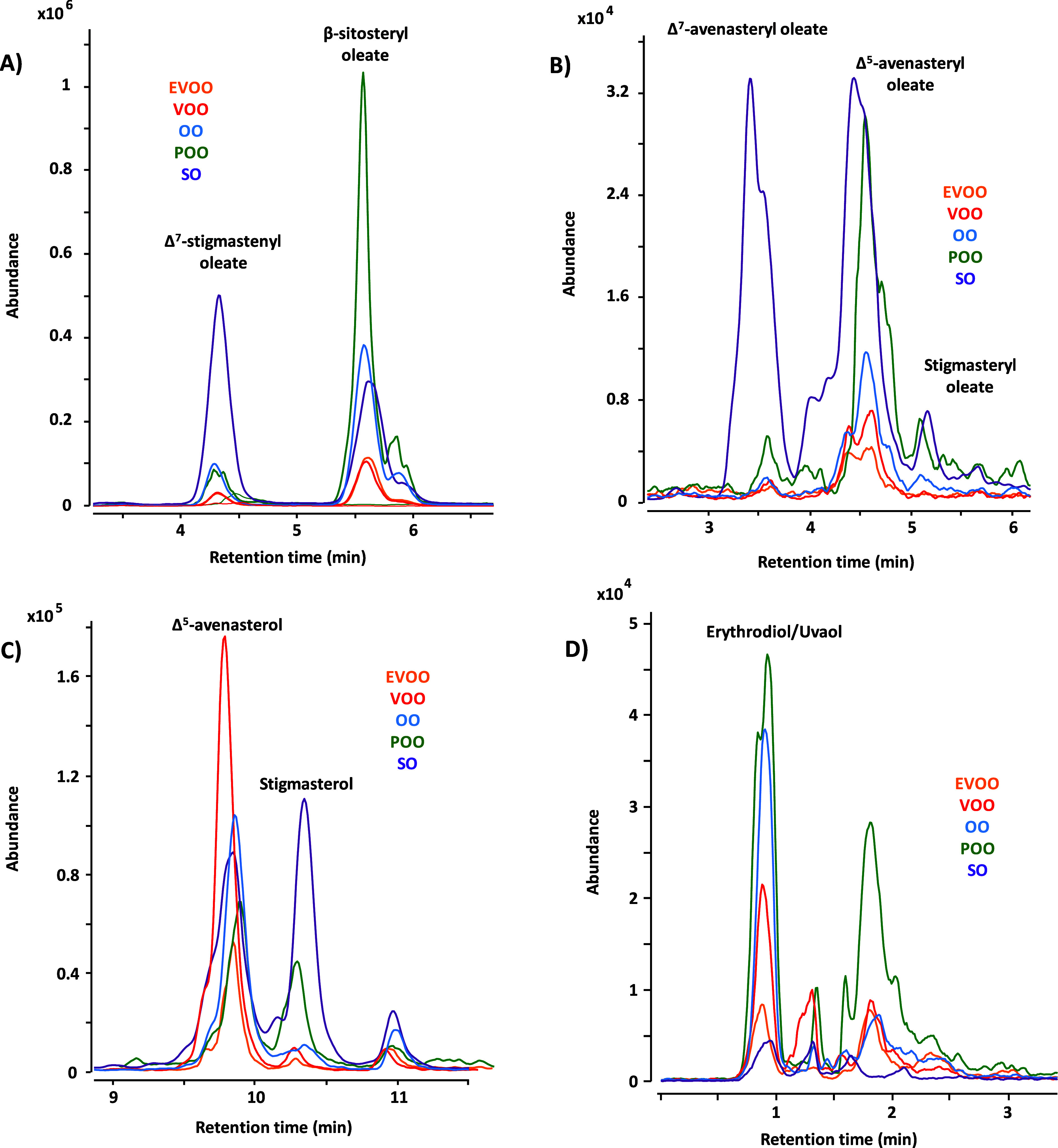
Comparison of the content of some of the most common sterols and triterpenic alcohols in a sample of EVOO (orange color), VOO (red color), OO (blue color), POO (green color) and SO (purple color) obtained by the 2D-LC-MS method. Overlay EICs for (A) Δ^7^-stigmastenyl oleate and β-sitosteryl oleate (*m*/*z* 398.3868; *m*/*z* corresponding to the presence of a ^13^C); (B) Δ^7^-avenasteryl oleate, Δ^5^-avenasteryl oleate and stigmasteryl oleate (*m*/*z* 395.3672); (C) Δ^5^-avenasterol and stigmasterol (*m*/*z* 395.3672); (D) erythrodiol and uvaol (*m*/*z* 425.3778). For details, see text.

Besides the total content of free and esterified sterols, some of the most common sterols of different oils can be used individually to extract useful information for authentication purposes. Some of the most representative sterols in olive and sunflower oils are shown in Figure 3. The esterified forms of Δ^7^-stigmastenol and Δ^7^-avenasterol are considered to be one of the most relevant diagnostic analytes for the detection of fraud in olive oils that are adulterated by sunflower oils.^37,38^ The higher concentration of these esters in SO compared to olive oil is confirmed by the results obtained in this study (Figure 3A,B). With regards to β-sitosterol, it is the most abundant sterol in both olive oil^5^ and SO.^39^Figure 3A shows the variation of β-sitosteryl oleate content among the different olive and SO categories, which agrees with the total ester content described above: OO, PO, and SO higher than EVOO and VOO.

Concerning Δ^5^-avenasterol, it is the second major sterol in olive oil and one of the most abundant in SO.^17^ As shown in Figure 3B,C this sterol was detected in all studied oils, both in the free and in the esterified form. Several articles reported an inverse correlation between Δ^5^-avenasterol and β-sitosterol levels in olive oils.^5^ This fact can be attributed to different reasons, including the effect of the stone/pulp weight ratio during olive ripening, as well as the role of Δ^5^-avenasterol as a precursor of β-sitosterol synthesis.^40^

Stigmasterol is another relevant sterol worth mentioning. An indicator of the authenticity of the oils analyzed was the absence of this sterol in the esterified fraction for EVOO and VOO samples (Figure 3B). Previous studies are consistent with the results obtained in this work and justify the detection of stigmasterol esters in EVOO or VOO as a consequence of the mixing of these oils with other vegetable oils.^23,24^ In addition, the increased content of free stigmasterol in olive oils of higher categories would also justify their possible adulteration with other refined olive oils or other vegetable oils.^37,39^ Another parameter to evaluate is the free campesterol/stigmasterol ratio, with an average value of 7.7 for EVOO, 13.7 for VOO, 5.1 for OO, 0.5 for POO, and 1.9 for SO. The results obtained in this study were consistent with other authors proposing a minimum value of 3 for the free campesterol/stigmasterol ratio in high-quality EVOO.^14,41,42^ Notably, a ratio below 3 was obtained for POO and SO, lower quality oils compared to EVOO or VOO.

In addition to sterols, the triterpenic alcohols content must also be considered. According to the regulation, the sum of erythrodiol and uvaol content in EVOO, VOO and OO cannot exceed 4.5% of the total sterol content, while this amount has to be higher than 4.5% in POO.^4^ Therefore, these two triterpenic alcohols are essential markers of adulteration of higher quality olive oil with POO. In this study, considering that all erythrodiol/uvaol content was in its free form, all samples of EVOO, VOO and OO complied with the standard. However, this content did not exceed 4.5% in the POO samples. This could be justified since only a semiquantitation of the free form of both dialcohols was done, but they can also occur as mono- and diesters of fatty acids.^43^ The quantification in its esterified form was not possible due to difficulties in their identification. However, Figure 3D shows a peak in all the olive oil samples around minute 2, with a higher intensity in the POO sample. Since this peak eluted within the range of the ester analysis, it may correspond to some erythrodiol or uvaol ester. Therefore, considering the content in free and esterified forms, the minimum established for POO could have been reached. However, the identification of these esterified compounds could not be confirmed. For sunflower oils, these triterpenic alcohols were found in lower concentrations in comparison to olive oils. This is consistent with the literature,^38^ being commonly used for authenticity purposes between olive and sunflower oils. Sum it all up, the method proposed in the present study has the remarkable advantage of providing additional data over the official procedure or methods published in the literature, where only the content of total sterols is measured. The method is streamlined as both saponification and derivatization steps are avoided, and the fat is analyzed without any chemical manipulation besides the SPE step. Knowing the original sterol structure has proved to be a valuable tool that, used in conjunction with other types of analysis, can contribute to the characterization and authentication of a wide variety of oil samples.

## Concluding Remarks

This work presents the development of a 2D-LC–MS method for the analysis of triterpenic alcohols, free and esterified sterols in oil samples. A RPLC–RPLC separation was performed using an ASM valve to avoid solvent incompatibility between both dimensions. Furthermore, the coupling of a selection valve to the 2D-LC setup was proposed to overcome the information loss limitation of the MHC interface. Therefore, an online heart-cutting method was developed with the advantage of acquiring the information from both dimensions by MS in a single file and in a short analysis time. The optimization of this methodology allowed the tentative identification and semiquantification of several sterols, both in free and esterified forms, in different categories of olive and sunflower oils. Another essential advantage of this approach is that it allows us to determine the total sterol content present in the oil, either in its free or esterified form, without having to resort to the time-consuming processes of saponification and derivatization, which are used in most of the applications described in the literature. In summary, the key benefits of this method in comparison to other approaches describes in literature are (i) the reduction of sample treatment steps; (ii) the simplification of 2D-LC-HRMS data analysis through the acquisition of both dimensions in a single analysis; and (iii) more exhaustive characterization of olive oil samples. To the best of our knowledge, this is the first time an online two-dimensional heart-cutting LC–MS method has been used to analyze the components of the unsaponifiable oil fraction. The ability to determine the distribution of these compounds in free or esterified fractions can be used as an effective tool for the thorough characterization of oils with similar sterol profiles and for assessing the authenticity of olive oil and detecting subtle fraud practices.

## Supporting Information Available

Additional information related to the synthesis of steryl ester standards, preliminary assays for ^1^D and ^2^D chromatographic optimization, sample treatment considerations, and analytical performance of the proposed 2D-LC-HRMS method (PDF)
