# Supplementary material for: Virgin Olive oil Authenticity Assays in a Single Run Using Two-Dimensional Liquid Chromatography-High Resolution Mass Spectrometry
Source: Anal Chem. 2024 Oct 15;96(43):17319–28. doi: 10.1021/acs.analchem.4c03678 (PMC11525930; doi:10.1021/acs.analchem.4c03678)
Supplement: Supplementary file 1 — ac4c03678_si_001.pdf [file ac4c03678_si_001.pdf]

Virgin olive oil authenticity assays in a single run using two-dimensional liquid chromatography-high resolution mass spectrometry

Irene Caño-Carrillo[1,2], Bienvenida Gilbert-López[1,2], Cristina Ruiz-Samblás[1,2], Antonio Molina-Díaz[1,2] and Juan F. García-Reyes[1,2]*

[1]Analytical Chemistry Research Group, Department of Physical and Analytical Chemistry, University of Jaén, Campus Las Lagunillas, 23071 Jaén, Spain
[2]University Research Institute for Olives Grove and Olive Oil, University of Jaén, Campus Las Lagunillas, 23071 Jaén, Spain

Table of contents

Text S1. Synthesis of steryl ester standards.

Text S2. Preliminary assays: simultaneous analysis of triterpenic alcohols, free and esterified sterols.

Text S3. Sample treatment considerations.

Table S1. Analytical performance of proposed 2D-LC-MS method for the analysis of triterpenic alcohols, free sterols and cholesteryl esters standards.

Figure S1. Proposed 2D-LC-MS setup. (A) Filling deck-A to collect the ^{1}D void volume fraction. (B) Simultaneous analysis of the ^{1}D and ^{2}D with selection valve in position 1 to acquire the ^{1}D effluent by MS. (C) Switching of the selection valve to position 2 at minute 8.5 to acquire the ^{2}D effluent by MS.

Figure S2. Results of the chromatographic optimization for the columns: (A) InfinityLab Poroshell 120 EC-18; (B) InfinityLab Poroshell 120 PFP; (C) SB-Phenyl; (D) Zorbax Eclipse Plus C_{18}. It was used a 1 mg L^{-1} concentration mixture of (1) fucosterol, (2) campesterol, (3) β-amyrin, (4) β-sitosterol, (5) cholestenol, (6) cholesteryl oleate, (7) cholesteryl palmitate.

Figure S3. Chromatograms resulting from the optimization of the ^{2}D separation for the columns (A) InfinityLab Poroshell 120 EC-18; (B) InfinityLab Poroshell 120 PFP; (C) SB-Phenyl. A mixture containing free sterols and triterpenic alcohols at 1 mg L^{-1} concentration was used: (1) erythrodiol, (2) uvaol, (3) brassicasterol, (4) cholesterol, (5) lupeol, (6) fucosterol, (7) Δ^5-avenasterol, (8) cholestenol, (9) campesterol, (10) stigmasterol, (11) β-amyrin, (12) Δ^7-stigmastenol, (13) β-sitosterol.

Figure S4. Evaluation of the retention of compounds in the filters tested. (A) Comparison of chromatograms obtained from a mixture of sterols and triterpene alcohols unfiltered and filtered with PTFE, nylon and cellulose. (B) Percentage losses for erythrodiol in the different filters. (C) Percentage losses for uvaol in the different filters. A mixture containing free sterols and triterpenic alcohols at 1 mg L^{-1} concentration was used: (1) erythrodiol, (2) uvaol, (3) brassicasterol, (4) cholesterol, (5) lupeol, (6) fucosterol, (7) Δ^5-avenasterol, (8) cholestenol, (9) campesterol, (10) stigmasterol, (11) β-amyrin, (12) Δ^7-stigmastenol, (13) β-sitosterol.

Figure S5. Comparison of the chromatogram obtained for the synthesized ester, the commercial standard and an oil sample for (A) stigmasteryl oleate; (B) β-sitosteryl oleate; (C) sitostanyl oleate.

Text S1*. Synthesis of steryl ester standards.*

Three steryl esters (β-sitosteryl oleate, stigmasteryl oleate and sitostanyl oleate) were prepared according to **Barnsteiner *et al.* (2012)**[1] with modifications in the initial reagent amount. The synthesis procedure was as follows: 0.05 mmol of free sterol and 0.10 mmol of fatty acid were heated in a nitrogen-flushed reaction vessel to 180 °C for 25 h. Afterwards, 2.5 mL KOH (1M) was added, and the esters were extracted three times with 2.5 mL of n-hexane/MTBE (3:2, v/v). Finally, the solvent was evaporated by a gentle nitrogen stream, and the residue was dried at 103 °C for 30 minutes. The remaining extract was redissolved in chloroform/methanol (1:1, v/v) and diluted before injection.

Text S2*. Preliminary assays: simultaneous analysis of triterpenic alcohols, free and esterified sterols.*

Experiments were carried out using a one-dimensional configuration (autosampler – column - MS) and a mixture of four free sterols, one triterpenic alcohol, and two steryl esters standards. The triterpenic alcohol eluted close to the free sterols in all conditions tested so that the discussion will focus on the separation of free and esterified sterols. All columns from 1 to 7 were initially evaluated with a MeOH/IPA gradient. With columns 1, 2, and 3, a good separation between free and esterified sterols was achieved. However, results showed that only free sterols were partially resolved, while both esters were co-eluted. This mobile phase was discarded for columns 4, 5, 6, and 7 since all compounds eluted in the void volume. The next step consisted of evaluating the different columns employing several mobile phase and gradient mixtures. The first combination involved the use of ACN/IPA in columns 1,6,7. Free sterols could not be completely resolved with column 1 (Zorbax Eclipse Plus C_{18}), while the separation of esters was successfully achieved under these conditions**.** On the other hand, neither free nor esterified sterols were retained in columns 6 and 7, and therefore, they eluted in the void volume.

Another combination was tested with columns 3, 5, 6, and 7 employing a mobile phase composed of (A) water acidified with 0.01% acetic acid and (B) acetonitrile, as described in **Zarrouk *et al*. (2010).**[2] Unacceptable peak shape and resolution were obtained with columns 3, 5, and 6 for esters and columns 3 and 5 for free sterols. Column 7 (SB-Phenyl) gave good results in terms of peak shape for both free and esterified sterols, although the separation was not satisfactory in any case. Only column 6 (Poroshell 120 PFP) offered acceptable separation for free sterols. Finally, columns 1, 5, 6,

and 7 were evaluated with H_2O/MeOH gradient elution (both acidified with 0.1% of formic acid). Esters could not be detected in columns 1 and 5, and their separation was also not possible under these conditions in columns 6 and 7. In contrast, column 5 (Poroshell 120 EC-C_{18}) and column 6 (Poroshell 120 PFP) offered appropriate performance for free sterols, obtaining for column 7 (SB-Phenyl) the best results in terms of resolution and peak shape compared to the other conditions evaluated for these compounds. Regarding the ester fraction, column 1 (Zorbax Eclipse Plus C_{18}) was the only column that separated them, employing ACN/IPA as the mobile phase **(Figure S2)**.

Text S3. ***Sample treatment considerations.***

The free and esterified sterol fractions were extracted with an SPE procedure described elsewhere.[3] However, in the last step of the process, different tests were performed to determine how the eluates were collected: (1) eluting the two fractions together, evaporating and reconstituting or (2) collecting the two fractions separately, evaporating, reconstituting and finally combining both extracts. The results of these experiments showed that cleaner extracts were obtained by collecting the fractions separately, as well as allowing easier solvent evaporation than if the 25 mL were collected in a single eluate. Another essential advantage of this procedure was the possibility of using a different dilution factor for each fraction, which was convenient since a lower dilution was required for the ^{1}D (1:10) than for the ^{2}D (1:100). Considering all these reasons, it was decided to collect the fraction of free and esterified sterols separately and subsequently combine them in the injection vial.

The filtration of the extracts before injection was another key aspect studied. Various filters (PTFE, nylon, cellulose) were tested with a mixture of free sterol and triterpenic alcohol standards. The signal obtained for the unfiltered standards was compared with the signal of the same compounds previously passed through the different filters **(Figure S4)**. A significant signal loss was observed for erythrodiol and uvaol, suggesting that these compounds were retained on the filter. Notably, for uvaol, a percentage loss of 70 % was calculated for all filters. For erythrodiol, a loss of 69% was obtained with the PTFE and cellulose filters, while the nylon filter retained the highest amount of analyte with a loss of 78%. For these reasons, unfiltered inject diluted extracts were injected.

Table S1. Analytical performance of proposed 2D-LC-MS method for the analysis of triterpenic alcohols, free sterols and cholesteryl esters standards.

Compound	Regression equation	Linearity (R^2)	RSD %		LOQ (ng μL^{-1})
			Intra-day (n = 3)	Inter-day (n = 6)	
Cholesteryl decylate	$y = 1.10 \cdot 10^3 x + 2.82 \cdot 10^2$	0.9901	6.2	19.2	0.1
Cholesteryl linolenate	$y = 9.98 \cdot 10^4 x + 2.83 \cdot 10^2$	0.9909	3.2	17.9	0.1
Cholesteryl linoleate	$y = 1.07 \cdot 10^3 x + 2.89 \cdot 10^2$	0.9912	6.9	19.6	0.1
Cholesteryl myristate	$y = 1.09 \cdot 10^3 x + 2.90 \cdot 10^2$	0.9904	5.4	13.5	0.1
Cholesteryl oleate	$y = 1.01 \cdot 10^3 x + 3.07 \cdot 10^2$	0.9912	0.4	15.5	0.1
Cholesteryl palmitate	$y = 8.29 \cdot 10^4 x + 2.45 \cdot 10^2$	0.9911	6.5	19.8	0.5
Cholesteryl stearate	$y = 3.16 \cdot 10^3 x + 1.97 \cdot 10^2$	0.9978	6.4	15.6	0.5
Cholesteryl erucate	$y = 3.06 \cdot 10^3 x + 2.36 \cdot 10^2$	0.9991	8.2	15.6	0.5
Erythrodiol	$y = 6.97 \cdot 10^4 x + 1.88 \cdot 10^3$	0.9905	8.0	13.4	0.1
Uvaol	$y = 6.97 \cdot 10^4 x + 1.88 \cdot 10^3$	0.9905	8.0	13.4	0.1
Brassicasterol	$y = 2.97 \cdot 10^4 x + 1.92 \cdot 10^3$	0.9979	6.4	18.3	0.01
Cholesterol	$y = 6.06 \cdot 10^4 x + 2.61 \cdot 10^3$	0.9977	7.6	19.6	0.01
Lupeol	$y = 5.88 \cdot 10^4 x + 2.87 \cdot 10^3$	0.9966	7.0	14.6	0.01
Fucosterol	$y = 1.76 \cdot 10^5 x + 3.76 \cdot 10^3$	0.9905	5.0	18.8	0.01
Δ^5-Avenasterol	$y = 1.76 \cdot 10^5 x + 3.76 \cdot 10^3$	0.9905	5.0	18.8	0.01
Cholestanol	$y = 2.85 \cdot 10^3 x + 8.02 \cdot 10^2$	0.9998	5.1	16.1	0.05
Campesterol	$y = 4.23 \cdot 10^4 x + 1.45 \cdot 10^3$	0.9935	4.7	14.4	0.01
Stigmasterol	$y = 1.82 \cdot 10^4 x + 1.19 \cdot 10^3$	0.9982	2.2	13.7	0.05
β-Amyrin	$y = 6.04 \cdot 10^4 x + 2.34 \cdot 10^3$	0.9955	5.3	14.4	0.01
Δ^7-Stigmastenol	$y = 2.35 \cdot 10^4 x + 7.36 \cdot 10^3$	0.9916	1.3	19.0	0.05
β-Sitosterol	$y = 5.31 \cdot 10^4 x + 1.76 \cdot 10^3$	0.9936	5.3	17.2	0.01

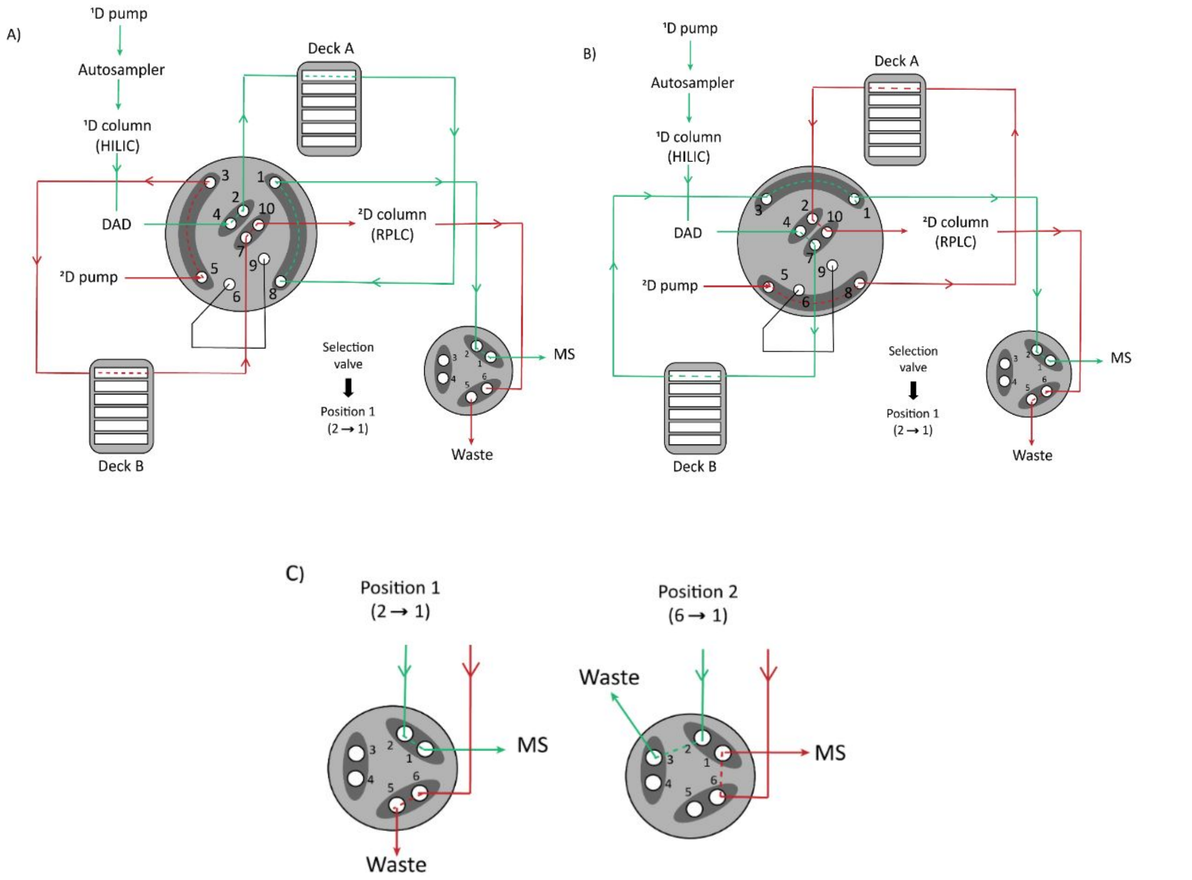

Figure S1. Proposed 2D-LC-MS setup. (A) Filling deck-A to collect the ^{1}D void volume fraction. (B) Simultaneous analysis of the ^{1}D and ^{2}D with selection valve in position 1 to acquire the ^{1}D effluent by MS. (C) Switching of the selection valve to position 2 at minute 8.5 to acquire the ^{2}D effluent by MS.

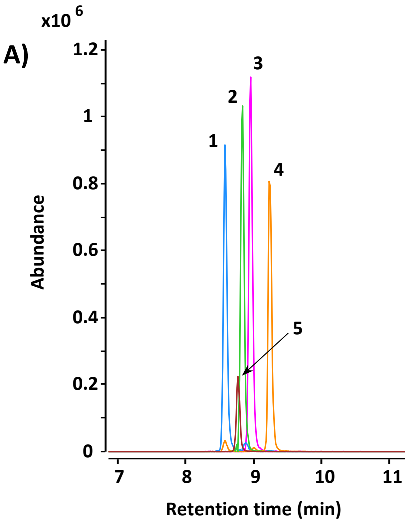

InfinityLab Poroshell 120 EC-18

t (min)	% B
0	90
10	100
13	100
13.1	90
15	10

A: H_2O + 0.1% FA
B: MeOH + 0.1% FA
F: 0.5 mL/min

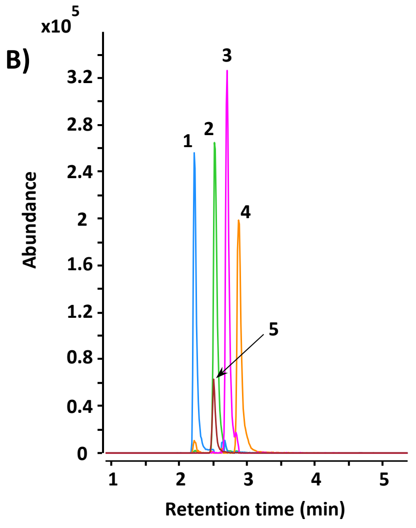

InfinityLab Poroshell 120 PFP

t (min)	% B
0	70
2	70
10	100
15	100
16	70
20	70

A: H_2O + 0.01% Acetic acid
B: ACN
F: 0.7 mL/min

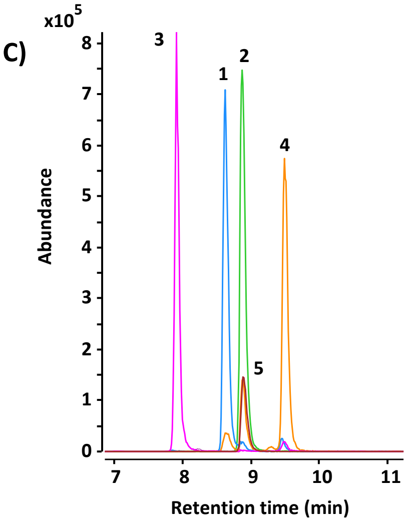

SB-Phenyl

t (min)	% B
0	80
20	100
23	100
23.1	80
25	80

A: H_2O + 0.1% FA
B: MeOH + 0.1% FA
F: 0.5 mL/min

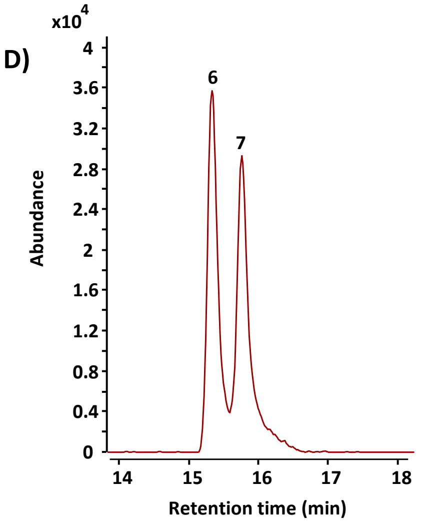

Zorbax Eclipse Plus C_{18}

t (min)	% B
0	50
15	75
20	75
20.1	50
25	50

A: ACN
B: IPA
F: 0.8 mL/min

Figure S2. Results of the chromatographic optimization for the columns: (A) InfinityLab Poroshell 120 EC-18; (B) InfinityLab Poroshell 120 PFP; (C) SB-Phenyl; (D) Zorbax Eclipse Plus C_{18}. It was used a 1 mg L^{-1} concentration mixture of (1) fucosterol, (2) campesterol, (3) β-amyrin, (4) β-sitosterol, (5) cholestenol, (6) cholesteryl oleate, (7) cholesteryl palmitate.

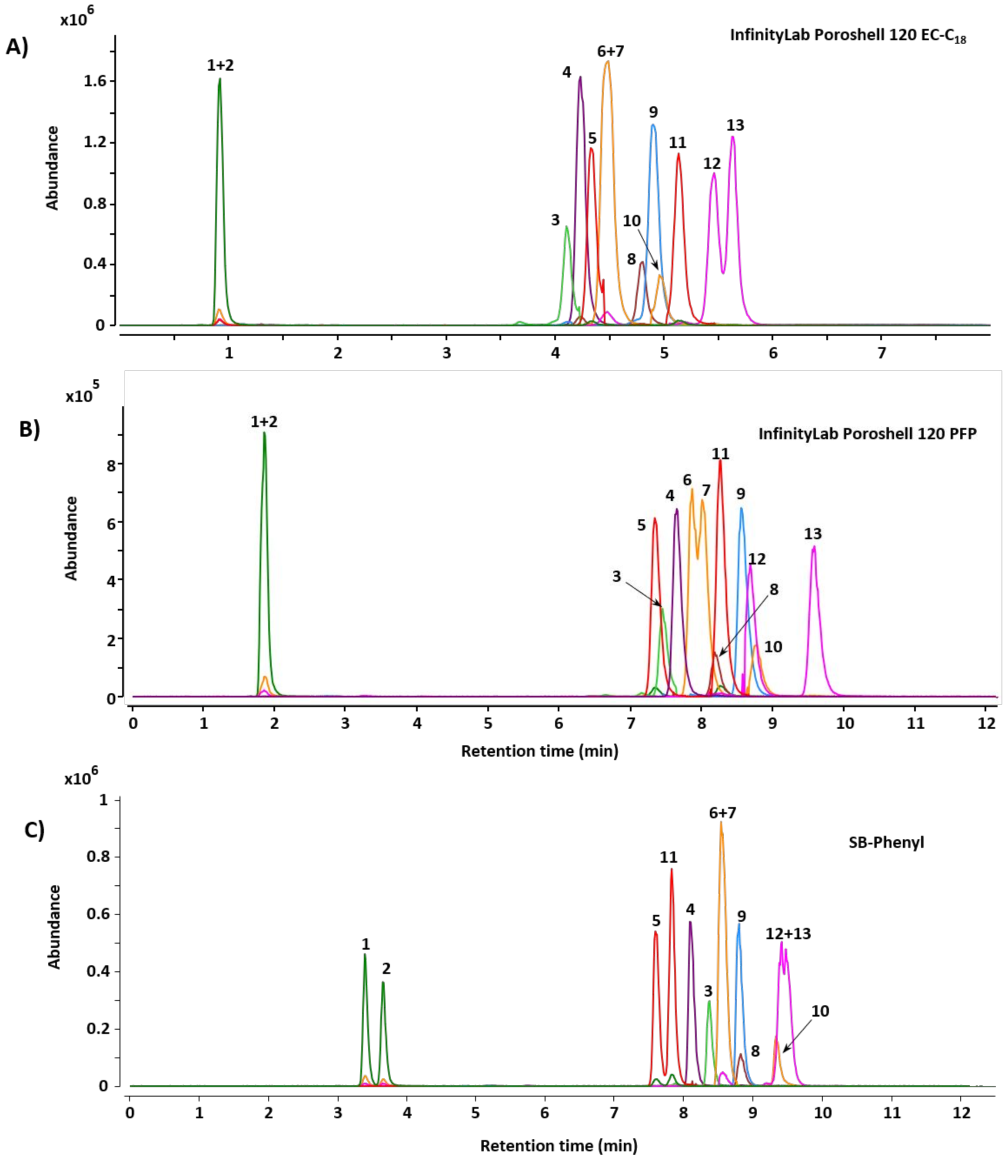

Figure S3. Chromatograms resulting from the optimization of the ^{2}D separation for the columns (A) InfinityLab Poroshell 120 EC-18; (B) InfinityLab Poroshell 120 PFP; (C) SB-Phenyl. A mixture containing free sterols and triterpenic alcohols at 1 mg L^{-1} concentration was used: (1) erythrodiol, (2) uvaol, (3) brassicasterol, (4) cholesterol, (5) lupeol, (6) fucosterol, (7) Δ^5-avenasterol, (8) cholestenol, (9) campesterol, (10) stigmasterol, (11) β-amyrin, (12) Δ^7-stigmastenol, (13) β-sitosterol.

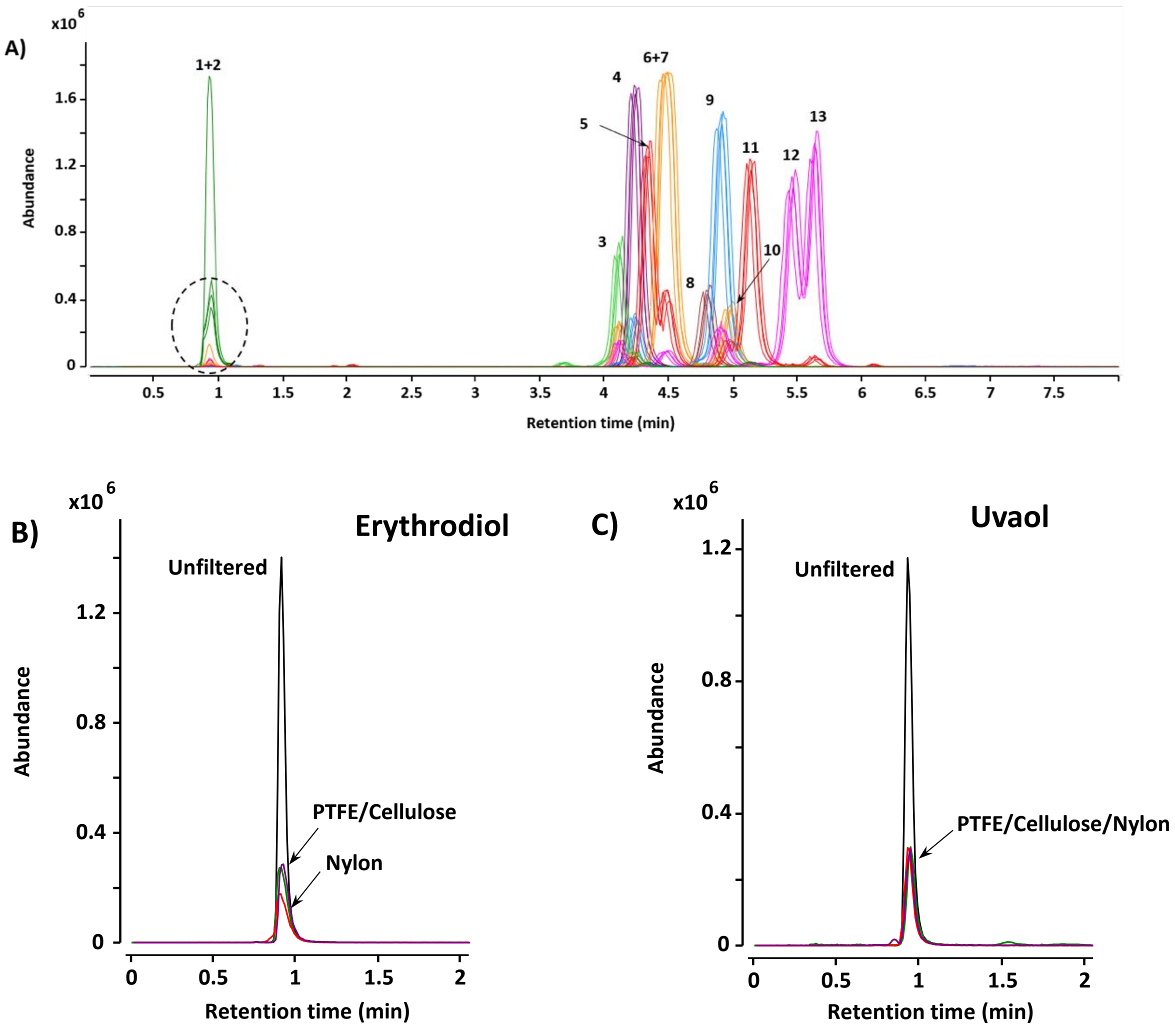

Filter	% Loss
PTFE (0.22 µm)	69
Nylon (0.22 µm)	78
Cellulose (0.45 µm)	69

Filter	% Loss
PTFE (0.22 µm)	70
Nylon (0.22 µm)	71
Cellulose (0.45 µm)	70

Figure S4. Evaluation of the retention of compounds in the filters tested. (A) Comparison of chromatograms obtained from a mixture of sterols and triterpene alcohols unfiltered and filtered with PTFE, nylon and cellulose. (B) Percentage loss for erythrodiol in the different filters. (C) Percentage loss for uvaol in the different filters. A mixture containing free sterols and triterpenic alcohols at 1 mg L^{-1} concentration was used: (1) erythrodiol, (2) uvaol, (3) brassicasterol, (4) cholesterol, (5) lupeol, (6) fucosterol, (7) Δ^5-avenasterol, (8) cholestenol, (9) campesterol, (10) stigmasterol, (11) β-amyrin, (12) Δ^7-stigmastenol, (13) β-sitosterol.

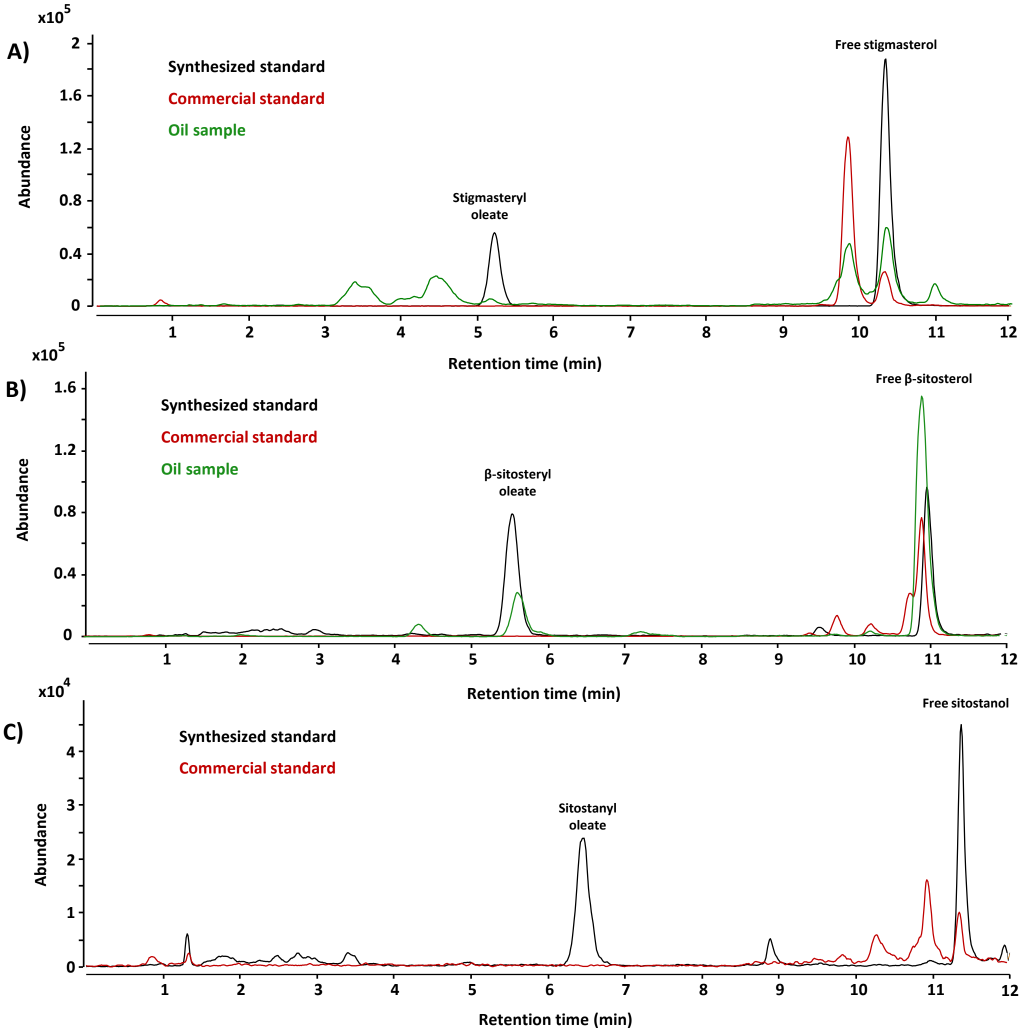

Figure S5. Comparison of the chromatograms obtained for the synthesized ester, the commercial standard and an oil sample for (A) stigmasteryl oleate; (B) β-sitosteryl oleate; (C) sitostanyl oleate.

---

1 Barnsteiner, A., Esche, R., di Gianvito, A., Chiavaro, E., Schmid, W., & Engel, K. H. (2012). Capillary gas chromatographic analysis of complex phytosteryl/-stanyl ester mixtures in enriched skimmed milk-drinking yoghurts. *Food Control*, 27, 275-283. http://dx.doi.org/10.1016/j.foodcont.2012.03.032

2 Zarrouk, W., Carrasco-Pancorbo, A., Segura-Carretero, A., Fernández-Gutiérrez, A., & Zarrouk, M. (2010). Exploratory Characterization of the Unsaponifiable Fraction of Tunisian Virgin Olive Oils by a Global Approach with HPLC-APCI-IT MS/MS Analysis. *Journal of Agricultural and Food Chemistry*, 58, 6418-6426. https://doi.org/10.1021/jf100024c

3 Cunha, S. S., Fernandes, J. O., & Oliveira, M. B. P. P. (2006). Quantification of free and esterified sterols in Portuguese olive oils by solid-phase extraction and gas chromatography–mass spectrometry. *Journal of Chromatography A*, 1128, 220-227. https://doi.org/10.1016/j.chroma.2006.06.039
